# Trajectories of MMSE and MoCA scores across the healthy adult lifespan in the Italian population

**DOI:** 10.1007/s40520-022-02174-0

**Published:** 2022-07-02

**Authors:** Edoardo Nicolò Aiello, Fabrizio Pasotti, Ildebrando Appollonio, Nadia Bolognini

**Affiliations:** 1 PhD Program in Neuroscience, School of Medicine and Surgery, University of Milano-Bicocca, Monza, Italy; 2 Department of Brain and Behavioural Sciences, University of Pavia, Pavia, Italy; 3 Neurology Section, School of Medicine and Surgery, University of Milano-Bicocca, Monza, Italy; 4 Department of Psychology, University of Milano-Bicocca, Milan, Italy; 5 Neuropsychological Laboratory, IRCCS Istituto Auxologico Italiano, Milan, Italy

**Keywords:** Mini-Mental State Examination, Montreal Cognitive Assessment, Aging, Cognitive screening

## Abstract

**Background:**

This study compares the performance at the Mini-Mental State Examination (MMSE) and Montreal Cognitive Assessment (MoCA) across the healthy adult lifespan in an Italian population sample.

**Methods:**

The MMSE and MoCA were administered to 407 Italian healthy native-speakers (165 males; age *range* 20–93 years; education range 4–25 years). A generalized Negative Binomial mixed model was run to profile MMSE and MoCA scores across 8 different age classes (≤ 30; 31–40; 41–50; 51–60; 61–70; 71–80; 81–85; ≥ 86) net of education and sex.

**Results:**

MMSE and MoCA total scores declined with age (*p* < 0.001), with the MoCA proving to be “more difficult” than the MMSE (*p* < 0.001). The Age*Test interaction (*p* < 0.001) indicates that the MoCA proved to profile a sufficiently linear involutional trend in cognition with advancing age and to be able to detect poorer cognitive performances in individuals aged ≥ 71 years. By contrast, MMSE scores failed in capturing the expected age-related trajectory, reaching a plateau in the aforementioned age classes.

**Discussion:**

The MoCA seems to be more sensitive than the MMSE in detecting age-related physiological decline of cognitive functioning across the healthy adult lifespan. The MoCA might be therefore more useful than the MMSE as a test for general cognitive screening aims.

**Supplementary Information:**

The online version contains supplementary material available at 10.1007/s40520-022-02174-0.

## Introduction

There is an overall favor among European [1], and especially Italian [2], healthcare practitioners towards the relevance of screening for cognitive impairment in asymptomatic adults within primary care settings, due to its beneficial entailments in respect to a timely intervention.

However, less consensus has been reached as to which test is most suitable to this aim [3]: the Mini-Mental State Examination (MMSE), despite being the most widespread cognitive screening test worldwide, has been questioned as to its feasibility in primary care [4], as being heavily subjected to ceiling effects [5] and thus scarcely sensitive to sub-clinical deficits [6]. By contrast, the Montreal Cognitive Assessment (MoCA), as widespread as the MMSE, has been highlighted as more sensitive than specific [7], and thus appropriate to detect even subtle cognitive changes when screening putatively healthy individuals [8, 9].

According to the World Health Organization (WHO), for a general-population screening program to be implemented, “[…] there should be a suitable diagnostic test that is available […]”, as well as “an agreed policy, based on respectable test findings and national standards […]” (http://www.euro.who.int/document/E88698.pdf). Hence, within the framework of cognitive screening, a first step for such requirements to be met would be to provide country-specific data on the performance on the MMSE and MoCA across the healthy adult lifespan, in order to explore their capability at detecting age-related, physiological changes in cognitive functioning and thus their feasibility for general-population screening aims.

To date, such an investigation has been only performed by Gluhm et al. [4], who showed that, in healthy English adults aged from 20 to 89 years, the MoCA was superior to the MMSE in cross-sectionally profiling involutional cognitive trajectories across age decades. Given that in Italy such data have not been provided yet [10], this study aimed at comparing MMSE and MoCA scores across the healthy adult lifespan in a large Italian population sample. Specifically, within this work, it was postulated that, in line with the findings of Gluhm et al. [4], the MoCA would outperform the MMSE as to the capability of detecting age-related physiological decline of cognitive functioning across the healthy adult lifespan also in the Italian population, based on the notion of the MoCA being “more difficult” to execute, and thus coming with a greater sensitivity when compared to the MMSE [7–9].

## Methods

The sample consisted in 407 Italian native-speakers (165 males, 242 females), with a mean age of 60.61 ± 13.74 years (range 20–93 years) and a mean education of 12.2 ± 4.42 years (range 4–25 years), who were recruited from different regions of Northern Italy. Sample stratification is shown in Supplementary Table 1. Participants had no history of (1) neurological/psychiatric disorders, (2) active psychotropic medications, (3) uncompensated, severe metabolic/internal conditions, (4) organ/system failures and (5) un-corrected vision and hearing deficits.

Participants were sub-divided into the following 8 age classes: ≤ 30; 31–40; 41–50; 51–60; 61–70; 71–80; 81–85; ≥86.

The Italian MMSE [11] and MoCA [12] were administered to every participant in a randomized order. To control for ceiling effects and high inter-individual variability in test scores (skewness and kurtosis values ≥ |1| and |3|, respectively) [13], a Negative Binomial mixed model was performed, by addressing the raw number of errors as the outcome [14], in order to test the Age*Test interaction (between-subject factor: *Age*; within-subject factor: *Test*) net of *Education* and *Sex*. *Subject* was addressed as the cluster, within which only a random intercept was fitted.

Significance level was set at *α* = 0.05 and multiple comparisons were Bonferroni-corrected. Analyses were run *via* SPSS 27 (IBM Corp., 2021).

## Results

The mean MMSE and MoCA scores for the whole sample were of 28.31±1.92 (range 20–30) and 25.62±3.84 (range 13–30), respectively. Error rates of participants on each test are shown separately for age classes in Table 1.

**Table 1:** Error rates on the MMSE and MoCA across age classes

	≤ 30 (*N* = 12)	31–40 (*N* = 9)	41–50 (*N* = 50)	51–60 (*N* = 173)	61–70 (*N* = 68)	71–80 (*N* = 49)	81–85 (*N* = 33)	≥ 86 (*N* = 13)	Significant comparisons within *Age*
MMSE (errors; *M* ± *SE*)	2.15 ± 1.03	0.76 ± .46	1.22 ± .52	1.78 ± .47	2.35 ± .95	1.91 ± .79	1.89 ± .8	1.99 ± .94	31–40 vs.61–70; 41–50 vs. 61–70; 51–60 vs. 61–70
MoCA (errors; *M* ± *SE*)	0.85 ± .46	2.83 ± 1.38	2.76 ± 1.13	3.75 ± 1.49	2.81 ± 1.14	6.15 ± 2.5	7.35 ± 3.02	9.19 ± 4	≤ 30 vs. 31–40, 41–50, 51–60, 61–70, 71–80, 81–85, ≥ 86; 31–40 vs. 71–80, 81–85, ≥ 86; 41–50 vs. 71–80, 81–85, ≥ 86; 51–60 vs. 71–80, 81–85, ≥ 86; 61–70 vs. 71–80, 81–85, ≥ 86
Significant comparisons within *Test*	–	–	MMSE < MoCA	–	MMSE < MoCA	MMSE < MoCA	MMSE < MoCA	MMSE < MoCA	

*MMSE* Mini-Mental State Examination, *MoCA* Montreal Cognitive Assessment; *N* number of participants for each age class

*p*-values refer to the Bonferroni-corrected decomposition of the Age*Test interaction within the Negative Binomial mixed model, thus addressing errors as the outcome

Both *Age* (*F*(7,796) = 8.52; *p *< 0.001) and *Test* (*F*(1,796) = 58.65; *p *< 0.001) main terms were significant, with error rates being overall higher on the MoCA when compared to the MMSE regardless of age, and linearly increasing on both tests with advancing age. As to covariates, *Education* was found to positively predict test scores (*F*(1,796) = 42.36; *p* < 0.001), whereas no *Sex* differences were detected (*F*(1,796) = 0.43; *p *= 0.514).

**Fig. 1:**
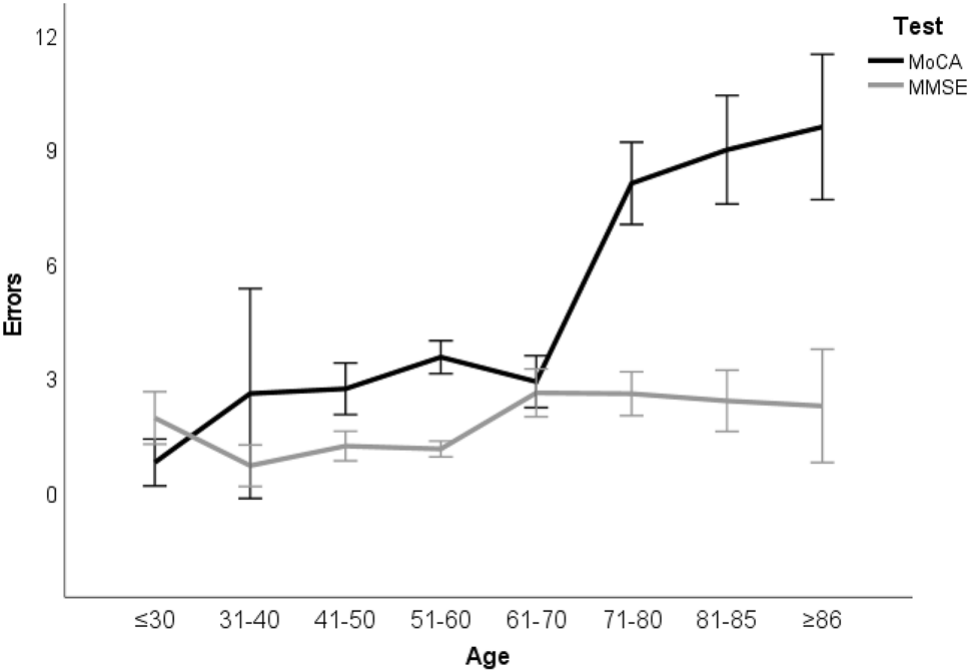
MMSE and MoCA errors across age classes. *MMSE* Mini-Mental State Examination, *MoCA* Montreal Cognitive Assessment. 95% CI are shown

The Age*Test interaction (net of education and sex), displayed in Fig. 1, was also significant (*F*(7,796)=8.38; *p* < 0.001). Its *post-hoc* decomposition (Table 1) revealed that: (1) the error rate on the MMSE was lower than that on the MoCA in participants aged 41–50 and for those aged ≥ 61; (2) error rates on the MMSE were lower for participants aged 31–60 when compared to those 61–70; (3) error rates on the MoCA for participants aged ≤ 30 were lower when compared to all other age classes, as well as those of participants aged 31–70 when compared to those aged ≥ 71. No other significant differences were detected.

Due to the significant main effect of *Education*, a further, specular model was run to test the Education*Test interaction, which instead addressed raw *Age* values and recoded *Education* (namely, ≤ 5, 6–11, 12–16 and ≥ 17 years). Net of other main effects that remained the same, such an interaction was significant too, with the error rate on the MoCA being higher than that on the MMSE in all *Education* classes (*p* ≤ 0.028). However, no significant comparisons yielded as to both the MoCA and the MMSE error rates across *Education* classes.

## Discussion

This study provides Italian clinicians and researchers with relevant information on the capability of the MMSE and MoCA in profiling age-related trends in cognitive functioning across the adult lifespan. Overall, the MoCA proved to be abler than the MMSE as to the detection of physiological cognitive changes with advancing age. Compliantly with WHO principles on general-population screening programs, the present Country-specific findings represent a first effort towards the identification of an adequate cognitive screening test to be administered to putatively normotypical, community-dwelling individuals. In this respect, consistently with the previous report by Gluhm et al. [4] in English healthy adults, the present results suggest that, at variance with the MMSE, the MoCA succeeds in capturing cognitive changes as a function of age, net of education and sex.

More specifically, the MoCA proved (1) to profile a sufficiently linear involutional trend in cognition with increasing age and (b) to be able to detect poorer cognitive performances in critical age classes, namely in individuals aged ≥ 71 years. By contrast, MMSE scores failed in capturing the expected age-related trajectory, but it also reaches a plateau in critical age classes. Notably, such a finding is in line with a recent Italian normative study on the MMSE [15] that showed that MMSE scores are overall similar, at a descriptive level, in healthy individuals aged from 70 to 94 years. It is thereupon reasonable to postulate that the MMSE would yield relatively low informativity as to age-related cognitive changes, and thus scarce utility for general-population screening aims, at variance with the MoCA.

In this last respect, it should be however noted that a recent meta-analysis by Nagaratnam et al. [16] suggests that the MMSE shows its highest capability in detecting cognitive decline among healthy individuals aged ≥ 84 years; however, this hypothesis cannot be confuted based on the present work since it includes a relatively small number of individuals falling under such an age range. Notably, Nagaratnam et al.’s [16] study also addressed ultra-centenary individuals, which were not represented in the present sample, despite their increase in number in the general population due to the overall higher life expectancy in Western countries. Future investigations should therefore focus MMSE performances in individuals in the eighth, ninth and tenth age decade.

This work also shows that, regardless of age, the MoCA is “more difficult” to execute than the MMSE, with such a discrepancy widening the most starting from the seventh age decade. This result is also supported by the fact that the error rates at the MoCA were systematically higher than that at the MMSE in all the education classes considered. Thereupon, findings herewith reported support the widespread notion [6] of the MoCA being more sensitive than the MMSE. Albeit such a feature is undoubtedly desirable for a screening test, it should be at the same time noted that the present data warn, in accordance with the literature [7], on the possibility that the MoCA may give rise to a higher false positive rates when compared to the MMSE. However, due to the absence of a third, independent gold-standard to explore the diagnostic accuracy of both tests, this work does not provide exhaustive information on the aforementioned possibility.

Besides those mentioned above, a number of further limitations should be finally listed: first, this study is cross-sectional, this limiting its external validity as to longitudinal inferences; second, age classes were partially inhomogeneous in size; third, the sample, although sufficiently well-stratified for age, education and sex, is region-specific.

In conclusion, the present work suggests that the MoCA is more sensitive than the MMSE in detecting age-related, physiological decline of cognitive functioning across the healthy adult lifespan in the Italian population. Such findings thus provide promising, albeit preliminary, evidence supporting the use of the MoCA, instead of the MMSE, as a test for general population-screening aims, in turn prompting future research aimed at confirming this hypothesis.

## Supplementary Information

Below is the link to the electronic supplementary material.

Supplementary file1 (DOCX 14 KB)

## Data Availability

Data analyzed within the present study are openly accessible at https://osf.io/8vr2s/.
