# Supplementary material for: Trajectories of MMSE and MoCA scores across the healthy adult lifespan in the Italian population
Source: Aging Clin Exp Res. 2022 Jul 2;34(10):2417–20. doi: 10.1007/s40520-022-02174-0 (PMC9637628; doi:10.1007/s40520-022-02174-0)
Supplement: Supplementary file 1 — Supplementary file1 (DOCX 14 KB) [file 40520_2022_2174_MOESM1_ESM.docx]

**Supplementary Table 1.** Sample stratification for age, education and sex.

|  | **Age (M/F)** | | | | | | | |
| --- | --- | --- | --- | --- | --- | --- | --- | --- |
| **Education** | **35≤** | **36-45** | **46-55** | **56-65** | **66-75** | **76-80** | **≥81** | **Total** |
| **5≤** | 0/0 | 0/0 | 0/0 | 4/1 | 3/11 | 4/11 | 4/12 | 15/35 |
| **6-8** | 1/0 | 1/0 | 4/14 | 13/10 | 7/5 | 3/4 | 4/4 | 33/37 |
| **9-13** | 4/4 | 4/3 | 21/39 | 30/35 | 8/6 | 0/6 | 6/9 | 48/102 |
| **14-16** | 1/2 | 0/1 | 3/9 | 3/5 | 0/0 | 0/0 | 0/1 | 7/18 |
| **≥17** | 2/1 | 1/4 | 10/16 | 22/21 | 1/2 | 0/1 | 1/5 | 38/50 |
| **Total** | 8/7 | 6/8 | 38/78 | 72/72 | 19/24 | 7/22 | 15/31 | 165/242 |
